# P-469. Evaluation of the HIV biobehavioral surveillance system for key populations in the Kyrgyz Republic, 2024

**DOI:** 10.1093/ofid/ofae631.668

**Published:** 2025-01-29

**Authors:** Akylai Kubatova, Nasyat Kemelbek kyzy, Aisuluu Kubatova, Dilyara Nabirova, Roberta Horth, Dinara Otorbaeva

**Affiliations:** Central Asia Advanced Field Epidemiology Training Program, Chuy, Kyrgyzstan; Central Asia Field Epidemiology Training Program, Bishkek, Kyrgyzstan, Bishkek, Bishkek, Kyrgyzstan; Ministry of Health of the Kyrgyz Republic, National Institute of Public Health, Bishkek, Kyrgyzstan, Bishkek, Bishkek, Kyrgyzstan; CDC Central Asia office, Almaty, Almaty, Kazakhstan; US Centers for Disease Control and Prevention, Dulles, Virginia; Department of Disease Prevention and State Sanitary and Epidemiological Supervision, Bishkek, Kyrgyzstan, Bishkek, Bishkek, Kyrgyzstan

## Abstract

**Background:**

New HIV infections are growing in Kyrgyzstan. Key populations (KP), including men who have sex with men (MSM), sex workers (SW), people who inject drugs (PWID), transgender people (TG), and labor migrants (LM) are disproportionately burdened by HIV. Biobehavioral surveillance is essential because routine HIV testing and treatment data in Kyrgyzstan does not adequately capture KP data due to stigma. A surveillance evaluation had not been conducted to identify system gaps and strengths.

**Table 1.**
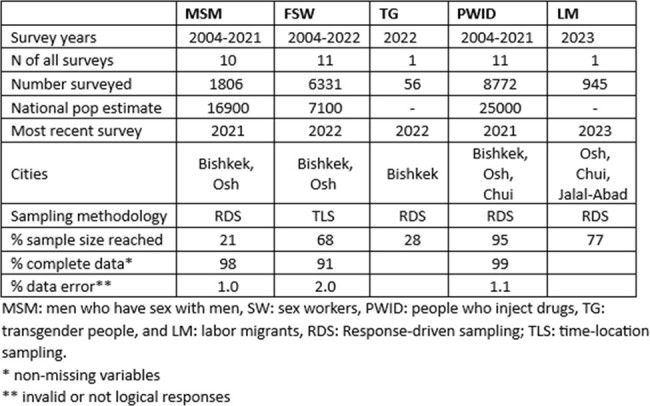
Summary of HIV biobehavioral surveys among key populations in the Kyrgyz Republic

**Methods:**

Using U.S. Centers for Disease Control and Prevention published guidelines for evaluating public health surveillance systems, we conducted an evaluation of Kyrgyzstan’s biobehavioral surveillance system from February to April 2023. We reviewed protocols, survey tools, data and reports from 2003 to 2023.

**Results:**

Biobehavioral surveys are conducted every 3 years. From 2003-2023, 34 studies were completed which included 1,806 MSM in 10 studies, 6331 SW in 11 studies, 8,772 PWID in 11 studies, 56 TG in 1 study and 945 migrants in 1 study. Population size estimations, which are useful for strategic planning, were 16,900 MSM, 7,100 SW, and 25,000 PWID. Protocols and tools were revised and sampling strategies updated before each survey. This helped ensure high quality standards and demonstrates the systems’ flexibility but limits the usefulness of data for comparing trends over time. Recent surveys for each KP failed to reach desired sample size; the TG study reached just 72% of target. Under-sampling, coupled with inherent bias introduced from the quasi-random sampling strategies used, may result in the data not being fully representative. Stability and timeliness were weaknesses; no surveys were conducted 2016-2021 due to funding shortages and the COVID-19 pandemic. Data quality was high. The recent SW survey (n=520) had high completeness of data (91%) and low error measured as invalid/not logical responses to questions (2%).

**Conclusion:**

The biobehavioral surveillance system for key populations in the Kyrgyz Republic is useful, flexible and of high quality. Important gaps in timeliness, stability, and representativeness could be addressed through stable financing for more surveys conducted at regular intervals.

**Disclosures:**

**All Authors**: No reported disclosures

